# Assessment of the occurrence of the second generation of *Mythimna loreyi* Duponchel (Lepidoptera: Noctuidae) using temperature-dependent developmental and oviposition models

**DOI:** 10.1371/journal.pone.0303841

**Published:** 2024-06-12

**Authors:** Sunghoon Baek, Min-Jung Kim, Eun Young Kim, Jin Kyo Jung, Chang-Gyu Park

**Affiliations:** 1 Department of Agriculture and Fisheries Convergence, Korea National University of Agriculture and Fisheries, Jeonju, Republic of Korea; 2 Forest Entomology and Pathology Division, National Institute of Forest Science, Seoul, Republic of Korea; 3 Crop Cultivation and Environment Research Division, National Institute of Crop Science, Rural Development Administration, Suwon, Republic of Korea; ICAR Research Complex for Eastern Region, INDIA

## Abstract

A significant crop pest, *Mythimna loreyi*, migrates annually to Korea and has been frequently observed in rice and corn fields. However, the phenology of this pest, particularly in relation to its ecological interactions and host crop seasons in Korea, remains poorly understood. This study aims to clarify the timing of the second generation of *M*. *loreyi* in Korea to enhance pest management strategies. To achieve this, we developed temperature-dependent models for developmental and ovipositional rates, studying these processes across five constant temperatures (15, 20, 25, 30, and 35°C). Our models, which showed a high correlation with observed data (*r*^2^ ≥ 0.93), include a theoretical approach that combines the developmental variation of immatures with the necessary degree-days for 50% egg laying and complete egg development. These predictions allow for the forecasting of the second generation’s occurrence, with relatively small deviations (one to three days) observed at two different field sites. The insights from this study are critical for both understanding the ecology of *M*. *loreyi* and for informing practical management decisions, such as optimal placement of barriers to prevent immigration and strategies for controlling local populations.

## Introduction

*Mythimna loreyi* is known to inhabit regions of Africa, as well as tropical or subtropical areas of India and Australia, along with certain parts of Asia [1]. This insect exhibits polyphagous behavior, posing a threat to various crops such as rice, wheat, maize, and others, with economic impacts depending on its population density [2, 3]. Although the status of the *M*. *loreyi* population in Korea remains uncertain, it is considered as a migratory insect that annually invades from other countries [1, 4, 5]. There is a high possibility that migrated populations could successfully complete additional generation in Korea [1, 4, 5]. While multiple generations per year have been reported in its native range [4] and in Korea [5], there is limited data on the phenology based on thermal responses, crucial information for effective pest management. This research gap suggests that *M*. *loreyi* may not yet be recognized as causing significant economic damage in its native regions.

However, there was a recent outbreak of this species in China, resulting in substantial economic losses [6–8]. This phenomenon suggests the potential for *M*. *loreyi* to become an invasive pest in non-native regions due to the absence of natural predators and the sensitivity of host plants to new invaders [9]. Furthermore, this insect has the ability to fly long distances for a long time [10]. These characteristics, combined with the ongoing climate change causing relatively milder winters, enhance the potential for *M*. *loreyi* to establish itself as an invasive species in non-native temperate regions. In Korea, the initial discovery of this species dates back to 1982 [11]. Following its first report, *M*. *loreyi* was still considered a relatively rare insect in Korea. However, since 2019, a significant number of *M*. *loreyi* individuals have been attracted to and captured in sex-pheromone traps designed for *Spodoptera frugiperda* [5]. Additionally, reports of crop damage caused by *M*. *loreyi* larvae in Korean corn fields have emerged in recent years, particularly in 2019 and 2020 [1]. These observations have led to suspicions about the establishment of the *M*. *loreyi* population in Korea. However, confirming its establishment remains challenging due to the elusive nature of Noctuidae moths and their strong flying capability [10, 12]. Recent advancements have facilitated indoor breeding methods for *M*. *loreyi* [1]. Conducting temperature-dependent experiments on *M*. *loreyi* is imperative to improve mass-rearing efficiency and predict its occurrence under field conditions.

Previous studies have investigated the temperature effects on the development of *M*. *loreyi* [12, 13], providing valuable insights into the lower developmental threshold temperature (LDT) and thermal requirements for eggs, larvae, and pupae of *M*. *loreyi*. While this information is valuable for understanding the fundamental ecology of *M*. *loreyi*, it is not enough to accurately predict its occurrence under field conditions. To predict its field occurrence, additional information is needed: the upper threshold temperature, optimal developmental temperature, developmental variation models, and oviposition models [14–18].

Predicting the field occurrence of migratory insects like *M*. *loreyi* is uncommonly complex, as their populations are influenced by variable weather conditions, particularly wind currents, in addition to their ecological traits. Nonetheless, accurate phenological forecasts are vital for the effective management of *M*. *loreyi* populations in Korea. In Korea, serious economic damage would be caused by second generations considering the occurrence patterns [1, 5], overwintering [2, 6, 7, 12], hosts [2, 3], and crop seasons in Korea. Moreover, it is impossible to accurately predict the occurrence of its first generation because they migrate from other countries [1, 4, 5].

Therefore, the primary objectives of this study were: (1) to develop linear and nonlinear developmental models, as well as survivorship models for *M*. *loreyi* immatures; (2) to establish oviposition-related models for its adult stage; (3) to develop its forecasting model applicable predict the second occurrence peak of *M*. *loreyi* in field conditions; and (4) to compare the differences between predicted and actual occurrences of *M*. *loreyi*.

## Materials & methods

### Experimental insects

The study commenced with the collection of *M*. *loreyi* larvae from corn fields located at the National Institute of Crop Science, Rural Development Administration, Republic of Korea (Latitude 37.274832, Longitude 126.984943). These larvae were individually reared in a petri-dish (90×15mm, SPL Life Sciences Co. Ltd.; Pocheon, Korea) by providing corn leaves until they reached adulthood. All individuals developed into adults, but the males died before females completed its development. Thus, male adults of *M*. *loreyi*, which attracted to sex-pheromone traps initially intended for *S*. *frugiperda*, were separately collected and introduced to the adult cage (Custom-made with transparent acryls, 260×310×310 mm) with the females. Although *M*. *loreyi* and *S*. *frugiperda* are distinct species, the male adults of *M*. *loreyi* were consistently attracted to the sex-pheromone traps intended for *S*. *frugiperda* [1, 4, 5, 19]. We carefully differentiated the males of *M*. *loreyi* from those of *S*. *frugiperda* by their distinct morphological features [5].

A cotton ball soaked in 10% sugar solution was supplied by hanging thread from the adult cage’s ceiling. To induce its oviposition, two sheets of paper towels were folded in half to create a gap, and then hung at both ends of the cage ceiling. Both paper towels and sugar solution were replaced daily to maintain optimal conditions. Eggs collected from the paper towels were then transferred to a plastic cage modified from a plastic container (Larval cage; 232×310×310 mm, LoknLock Co.; Seoul, Korea) by attaching a wire mesh (200 mesh, 70×90 mm) on the cage lid for ventilation and respiration.

When the larvae hatched within the plastic cage, they were provided with a meridic diet [1, 19]. Upon completing their larval development, pupae were transferred to adult cages. Starting from the third generation, the larvae of *M*. *loreyi* were reared both individually and in groups. Each larval cage housed approximately 100 individuals, and two such cages were used for group rearing. Additionally, over 200 individual larvae of *M*. *loreyi* were reared in petri dishes (90×15 mm). Pupae from both rearing systems were randomly allocated to two adult cages. Prior to the start of the experiment, ten generations of *M*. *loreyi* had been reared in the insect room under controlled conditions (25 ± 2°C, 50 ± 10%, 14:10 h (L:D)).

### Temperature dependent experiment procedures

Experiments were conducted under a photoperiod of 16:8 (L:D) h with a RH of 50–80% in an environmental chamber (DS-8CL, Dasol Scientific; Hwaseong-si, Korea). Before starting the experiment, the temperature of each chamber was monitored to ensure they reached the desired mean temperatures of 15, 20, 25, 30, and 35°C, using temperature loggers (U12-012, OnSet Computer Corp.; Pocasset, MA, USA). All temperatures, except for 35°C (± 2°C), were maintained within 0.5°C of the mean. At 35°C, temperature stabilization delays occurred following the turning on and off chamber lights.

The experimental population originated from a group maintained under laboratory conditions. We obtained an egg cohort for each temperature by inducing mating and oviposition under corresponding conditions. To ensure fertilization, pairs of same-aged adults (<1 day old) were placed in plastic insect cages (#310075, SPL Life Sciences Co. Ltd.) for each temperature condition. Each cage was equipped with a cotton ball soaked in a 10% sugar solution and a folded paper towel for oviposition. Eggs deposited on the paper towels were collected daily and transferred to larva cages set at the respective chamber temperatures. Due to incomplete copulation at 35°C, eggs (<1 day old) oviposited at 30°C were used for the egg development experiment at 35°C. Egg hatching at each temperature was monitored and recorded daily. The monitoring was conducted until no additional hatching occurred for seven consecutive days from the last hatch.

For the larval development experiment, 90 newly hatched larvae (< 1 day old) were randomly selected and individually transferred into insect rearing cups (#9091, Frontier Agricultural Sciences; Newark, USA) at each temperature. At 35°C, 90 individuals (< 1 day old) that hatched at 30°C were used again because no eggs successfully hatched at this higher temperature. Each larva was provided with one piece (approximately 4g) of artificial diet. Larval development and survival were checked daily, and the food was replaced before it dried out or showed signs of fungal contamination.

Once individual larvae at experimental temperatures developed into pupae, the sex of each pupa (three-day-old) was identified and marked on the rearing cup. The pupal development experiment continued at each temperature without introducing new individuals, except at 35°C. For this temperature, 90 pupae (<1 day old) reared at 30°C were used, compensating for the absence of viable pupae from the 35°C environment.

The oviposition experiment was conducted under four temperature conditions (15, 20, 25, and 30°C), as no individuals completed their development at 35°C. Newly emerged adult pairs (< 1 day old) were placed in cages (#310075), each equipped with a cotton ball soaked in 10% sugar solution and a folded paper towel to serve as an oviposition substate. We monitored twenty pairs at each temperature. Females were observed until their demise, at which point the experiment for that cage concluded. Males were maintained separately, with replacements made for any that died before the end of the female’s oviposition period. Adult survival and egg counts were documented daily, and the cotton balls and paper towels were regularly refreshed.

### Data analysis and model development

Preliminary data analysis, including normality and variance homogeneity of developmental and ovipositional data, was conducted with the Shapiro-Wilk test and Bartlett test in SAS [20]. ANOVA was applied to examine the effects of temperature on development and oviposition of *M*. *loreyi* using PROC GLM in SAS [20]. Tukey HSD test [20] and Bonferroni correction were applied for mean separation.

The reciprocal of developmental times (in days), representing developmental rates for each stage (i.e., egg, larva, pupa, and immature) of *M*. *loreyi*, was modeled using both linear and non-linear approaches [21, 22]. For the linear model, data only from the linear portion of each stage were regressed against temperature using PROC REG in SAS [20]. Data from the 35°C condition were excluded from these analyses because they did not align with the linear portion of the dataset. The linear model equation was:

r(T)=ax+b (1)

where *r*(*T*) was the developmental rate at temperature *T* (°C), *a* was the slope, and *b* was the y-intercept. The LDT and thermal constant (in degree-days) were estimated as–*a* / *b* and 1 / *a*, respectively [23, 24].

In the non-linear model, all developmental data were used to fit developmental rate against temperature using PROC NLIN in SAS [20]. Among available multiple models, Briére equation [22] was applied because this model was applicable to all developmental stages of *M*. *loreyi* in this study and there was no problem in the model verification (e.g., *P* value, the relationships between each parameter and its standard error of mean, *r*^2^ value, the biological meaning of each parameter such as optimal temperature and upper developmental threshold) and model validation. The applied equation [22] was:

$$r\left(T\right)=S\times T(T-T_{min}){\left(T_{max}-T\right)}^{1/2}$$ (2)

where *r*(*T*), *S*, *T*_*min*_, and *T*_*max*_ were the developmental rate at temperature *T* (°C), an empirical constant, the LDT, and the upper developmental threshold temperature, respectively.

The developmental variation model for each life stage of *M*. *loreyi* was established using the cumulative proportion of completed development. The developmental time for each stage was determined by multiplying the number of days required for development with the average daily development rate observed at each temperature. These standardized physiological ages were transformed as degree-days by multiplying each physiological age with a thermal constant estimated from the linear developmental model at each temperature. The developmental variation models of *M*. *loreyi* were fitted to the Weibull function:

$$P[DD(px)]=1-e^{-{\left[DD(px)/\alpha \right]}^{\beta }}$$ (3)

where *P*[*DD*(*p*_*x*_)] was the cumulative proportion of individuals that completed their development at transformed physiological time (*DD*(*p*_*x*_)), and *α* and *β* were fitted parameters in PROC NLIN of SAS [20].

In survivorship models, survivorship (%) was calculated as the number of individuals divided by the initial number of tested individuals. The survivorships of each developmental stage against experimental temperatures were described by a nonlinear equation selected from the library of TableCurve [25].

$$s(T)=100-e^{-(\alpha +\beta T+\gamma T^{2})}$$ (4)

where *s*(*T*) was the survivorship (%) at temperature *T* (°C), and *α*, *β*, and *γ* were fitted parameters in PROC NLIN of SAS [20].

The adult developmental rate model for *M*. *loreyi* is analogous to the developmental model used for its immature stages, with the distinction that adult longevity is accounted for as a phase of adult development. Thus, the reciprocal of mean longevity (in days) of adults was fit to a linear model [21] and a nonlinear model selected from the library of TableCurve [25]. The parameters of the linear model (Eq 1) were estimated with PROC REG in SAS [20]. The selected non-linear model was:

$$r\left(T\right)=A+BT^{2}+CT^{3}$$ (5)

where *r*(*T*) was the developmental rate at temperature *T* (°C), *A*, *B*, and *C* were fitted model parameters.

Fecundity model of female adult was developed by fitting the mean total number of eggs oviposited per female adult at each temperature to the Briére function (Eq 2). To prevent possible confusion between the developmental rate model of immatures and the fecundity model of female adults, the parameter *S* of immature model was expressed as *n* in adult model. The parameters were estimated in PROC NLIN of SAS [20].

Both oviposition rate and survival rate models used the physiological time of adult females as the dependent variable. The physiological time in both models was calculated by multiplying adult developmental time (days) with the average of daily developmental rate at each temperature. The cumulative proportion of oviposited eggs by a female adult until physiological time (ovipositional rate model) was expressed with the Weibull function:

$$O(p_{x})=1-e^{-{\left(\frac{p_{x}}{\alpha }\right)}^{\beta }}$$ (6)

where *O*(*p*_*x*_) was the cumulative proportion of oviposited eggs at a physiological time (*p*_*x*_) of a female adult, and *α* and *β* were fitted parameters in PROC NLIN of SAS [20].

The survival rate (%) at the physiological time of female adults was calculated by dividing the number of survived adults at a given physiological time with the initial number of tested adults. The equation was:

$$S(p_{x})=100e^{-{\left[(\frac{p_{x}}{\gamma }\right)}^{\delta }]}$$ (7)

where *S*(*p*_*x*_) was the percentage of live female adults at a physiological time (*p*_*x*_), *γ* was the physiological time of 50% survival, and *δ* was fitted parameters. The parameters were estimated in PROC NLIN of SAS [20].

### Monitoring of adult occurrence at fields

The occurrence of *M*. *loreyi* was observed at four rice fields located in Anseong-Si (N 37.014942, W 127.303215), Suwon-Si (N 37.262972, W 126.987966), Shinan-Gun (N 34.8461, W 126.3576), and Namhae-Gun (N 34.82552, W 127.8872) from April to November in 2022. All fields were nationally managed and controlled observational rice fields by the Rural Development Administration of Korea to monitor agricultural pests and estimate harvest amounts. One cone trap (Gempler’s Inc.; Janesville, WI, USA) was placed at the edge of each field. The trap was baited with a synthetic sex-pheromone blend (#50236, Pherobank; Broekweg, Netherlands). The lures were replaced every four weeks. The male adults captured in the trap were brought to the laboratory and counted weekly.

### Forecasting model development and validation

Forecasting models for the second occurrence peak of *M*. *loreyi* at fields were developed using the results of temperature-dependent development and oviposition in this study. Degree-day accumulation for forecasting model was planned to start from the first peak of adult occurrence at fields because first peak population of *M*. *loreyi* could have migrated from other countries to Korea. Thus, the model was developed by shifting the developmental variation model of *M*. *loreyi* immatures with the required degree-days of 50% egg laying by female adults and egg developmental completion.

To compare the timings between the developed forecasting model and actual occurrence of *M*. *loreyi* at fields, the first peak time of caught adults at a trap was bio-fixed at each site. Daily degree-days were calculated with the excel sheet provided by UC IPM Online (http://imp.ucanr.edu) with the single-sine method. The daily maximum and minimum temperature data were obtained from the weather stations of the Korea Meteorological Administration (http://www.kma.go.kr) closest to each site. For the calculation of accumulated degree-days, the LDTs was set at 11.25°C up to 72.5 degree-days (DD) to cover the thermal requirement for 50% oviposition. From 72.5 DD to 128.1 DD, which encompasses the thermal requirement for 50% oviposition plus egg development, the LDT was 12.9°C. After 128.1 DD, which is for the immature development phase, the LDT used was 10.8°C. The upper developmental threshold for the calculation was 35.0°C for whole periods, but any daily maximum temperatures did not reach to the upper developmental threshold of *M*. *loreyi*. Cumulative proportional data for caught adults were constructed using trap data of second occurrence peaks at each site. The period of the second occurrence peak was determined by the increase in individuals caught in the traps after the first peak to the successive decrease in catches.

## Results

### Temperature-dependent experiment

**Fig 1:**
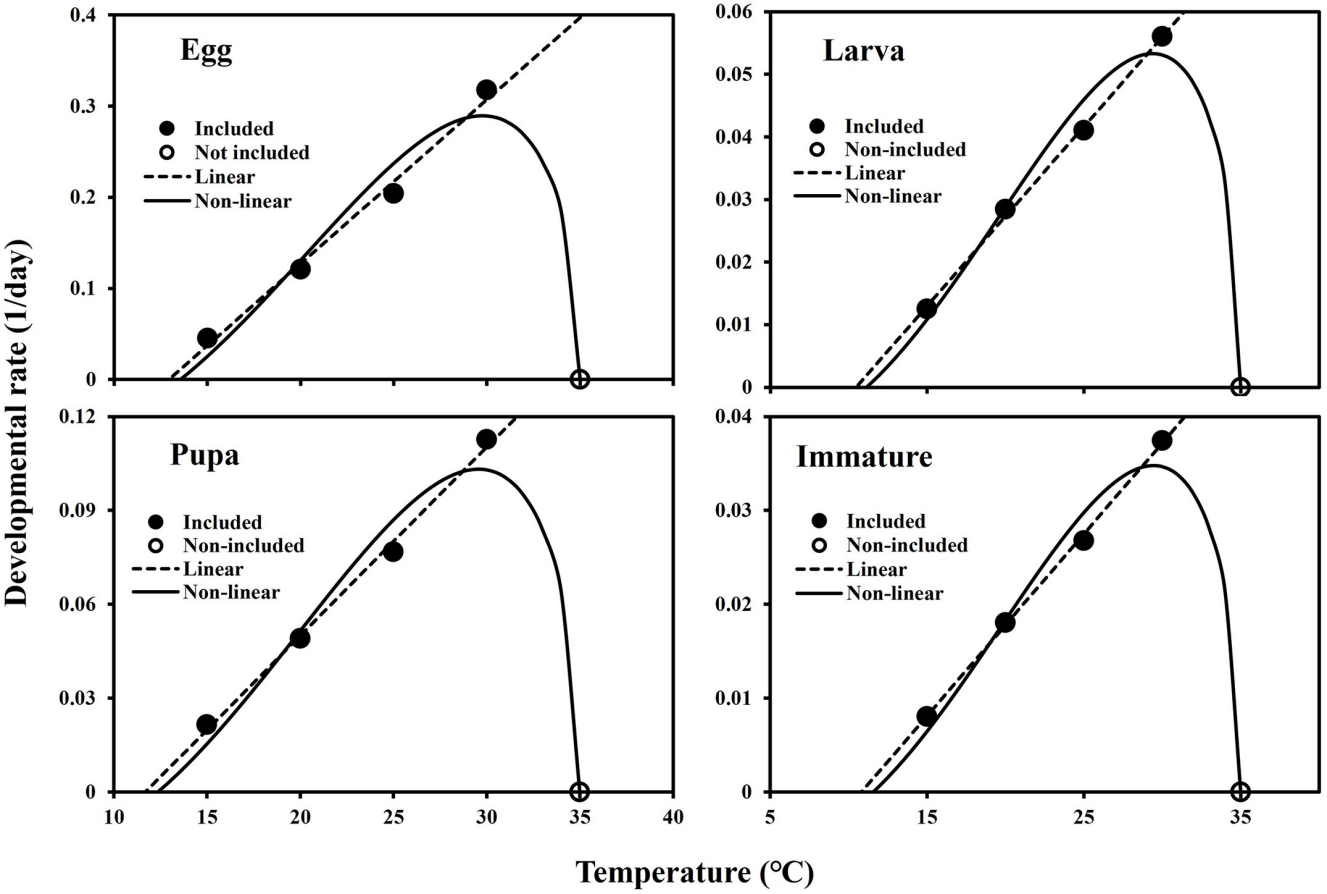
Developmental rate models of each developmental stage of *M*. *loreyi* according to constant temperature (°C).

**Fig 2:**
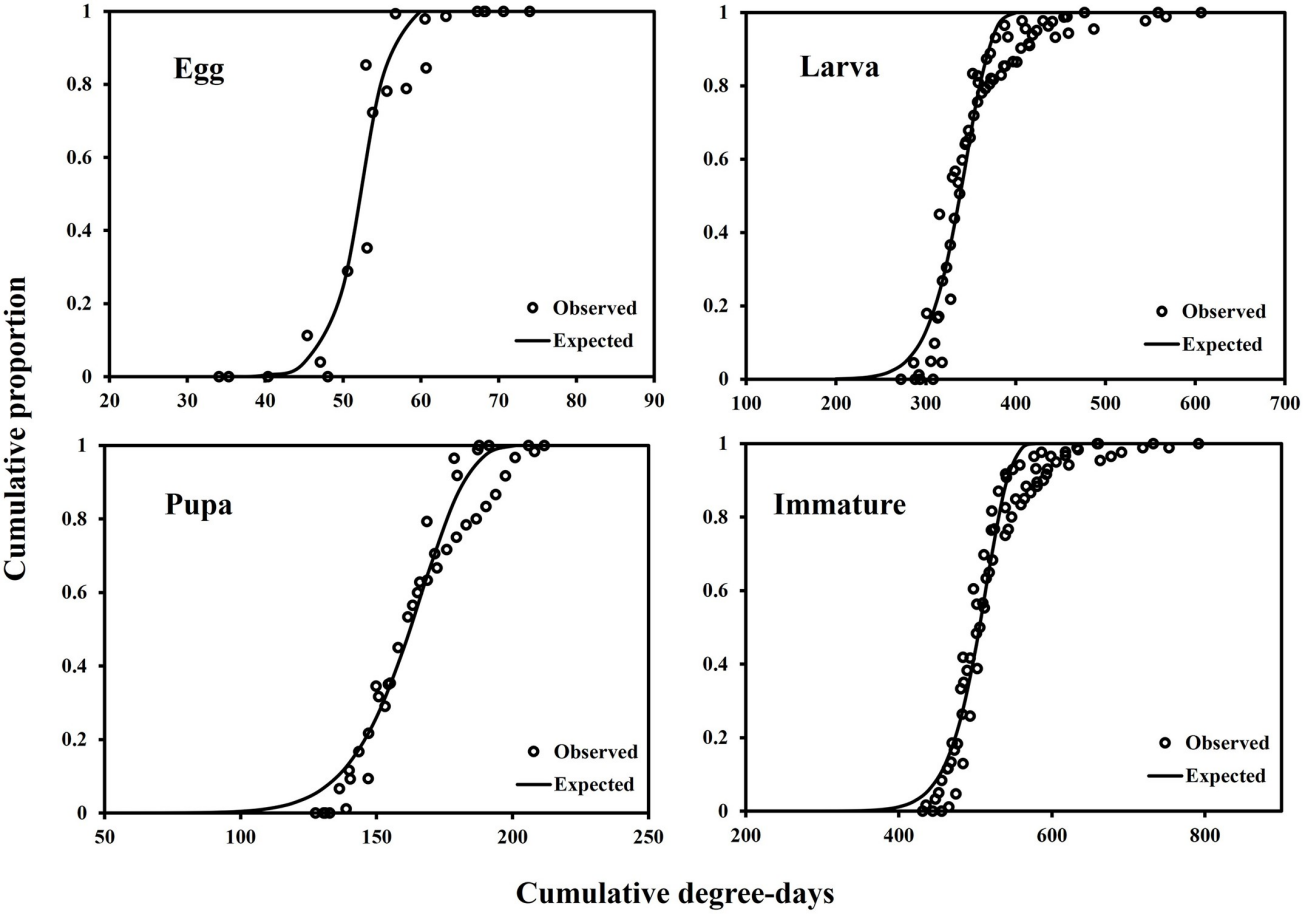
Survivorship models of each developmental stage of *M*. *loreyi* at constant temperature (°C).

The eggs, larvae, pupae, and immatures (from egg hatching to adult emergence) of *M*. *loreyi* could complete their development from 15 to 30°C, but no individuals at 35°C could (Table 1). The developmental rate of each developmental stage also increased from 15 to 30°C (Fig 1, Table 1). However, the temperature effects on survivorship were different according to its developmental stage. The effect was not clear in its larval stage, but the survivorship of eggs was the highest at 20°C among all experimental temperatures (Fig 2, Table 1).

**Table 1:** Developmental time (day, mean ± SD) at constant temperature (°C).

Temperature (°C)	Egg	Larva	Pupa	Immature^1^
15	22.0 ± 1.76 a^2^ (142 / 2,472) ^3^	80.0 ± 9.75 a (82 / 90)	46.3 ± 5.76 a (60 / 82)	124.4 ± 11.80 a (60 / 90)
20	8.3 ± 0.56 b (9,532 / 10,887)	35.1 ± 2.57 b (87 / 90)	20.4 ± 1.53 b (85 / 87)	55.4 ± 3.26 b (85 / 90)
25	4.9 ± 0.33 c (13,169 / 15,630)	24.3 ± 4.17 c (89 / 90)	13.0 ± 1.03 c (86 / 89)	37.3 ± 4.11 c (86 / 90)
30	3.2 ± 0.35 d (3,430 / 6389)	17.8 ± 2.26 d (90 / 90)	8.9 ± 0.76 d (87 / 90)	26.7 ± 2.64 d (87 / 90)
35	-^4^ (0 / 432)	- (0 / 90)	- (0 / 90)	- (0 / 90)

^1^ Immature period indicates the period from first instar to adult emergence.

^2^ Means within a column followed by the same letter are not significantly different (*P* > 0.05; Tukey’s HSD test at 95% confidence intervals).

^3^ Numbers in parentheses indicate the numbers of individuals that survived / total number of individuals tested.

^4^ No individuals survived.

The developed linear models showed good fits to the linear portions of developmental rate data of each developmental stage of *M*. *loreyi*: egg (*F* = 211.6; df = 1, 2; *P* = 0.005), larva (*F* = 1,086.4; df = 1, 2; *P* < 0.001), pupa (*F* = 434.5; df = 1, 2; *P* = 0.002), and immature (*F* = 1,575.3; df = 1, 2; *P* < 0.001) (Fig 1, Table 2). From the linear model, the estimated LDT and thermal constant were approximately 12.9°C and 55.6 degree-days (DDs) for eggs, 10.5°C and 348.9 DDs for larvae, 11.7°C and 166.1 DDs for pupae, and 10.8°C and 515.7 DDs for immatures, respectively.

**Table 2:** Parameters (estimated parameters ± SEM) of linear developmental models of *M*. *loreyi*.

Stage	Parameters		*r*^2^ value
	*a*	*b*	
Egg	0.017999 ± 0.0012374	-0.2328 ± 0.02869	0.99
Larva	0.002866 ± 8.70×10^−5^	-0.0300 ± 0.00202	0.99
Pupa	0.006019 ± 0.0002887	-0.0704 ± 0.00669	0.99
Immature^1^	0.001939 ± 4.89×10^−5^	-0.0210 ± 0.00113	0.99

^1^ Immature period indicates the period from first instar to adult emergence.

The non-linear models were also well-fit to the developmental rate: egg (*F* = 26.0; df = 2, 2; *P* = 0.037), larva (*F* = 55.3; df = 2, 2; *P* = 0.018), pupa (*F* = 31.9; df = 2, 2; *P* = 0.030), and immature (*F* = 45.1; df = 2, 2; *P* = 0.022) (Fig 1, Table 3). From the non-linear model, the estimated optimal and upper developmental temperatures (°C) were approximately 29.8 and 35.0 for eggs, 29.4 and 35.0 for larvae, 29.6 and 35 for pupae, and 29.4 and 35.0 for immatures, respectively.

**Table 3:** Parameters (estimated parameters ± SEM) of non-linear developmental models of *M*. *loreyi*.

Stage	Parameters			*r*^2^ value
	*S*	*T*_*min*_	*T*_*max*_	
Egg	2.62×10^−4^ ± 4.53×10^−5^	13.5510 ± 1.80069	35.0 ± 0.00013	0.96
Larva	4.20×10^−5^ ± 5.50×10^−6^	11.1505 ± 1.65615	35.0 ± 0.00009	0.98
Pupa	8.70×10^−4^ ± 1.44×10^−5^	12.3616 ± 1.88338	35.0 ± 0.00011	0.97
Immature*	2.80×10^−5^ ± 4.00×10^−6^	11.5491 ± 1.74864	35.0 ± 0.00010	0.98

* Immature period indicates the period from the first instar to adult emergence.

Survivorship models accurately described the changes in survivorship of *M*. *loreyi* according to experimental temperatures: egg (*F* = 11.8; df = 2, 2; *P* = 0.048), larva (*F* = 674.5; df = 2, 2; *P* = 0.001), pupa (*F* = 1,259.7; df = 2, 2; *P* < 0.001), and immature (*F* = 390.3; df = 2, 2; *P* = 0.003) (Fig 2, Table 4).

**Table 4:** Parameters (estimated parameters ± SEM) of survivorship models of *M*. *loreyi*.

Stage	Parameters			*r*^2^ value
	*α*	*β*	*γ*	
Egg	13.030 ± 2.6253	-0.813 ± 0.2444	0.016 ± 0.0049	0.93
Larva	27.806 ± 14.3347	-2.491 ± 1.3634	0.052 ± 0.0273	0.99
Pupa	22.175 ± 2.3599	-1.827 ± 0.2241	0.038 ± 0.0045	0.99
Immature*	20.775 ± 2.9593	-1.668 ± 0.2807	0.034 ± 0.0056	0.99

* Immature period indicates the period from the first instar to adult emergence.

**Fig 3:**
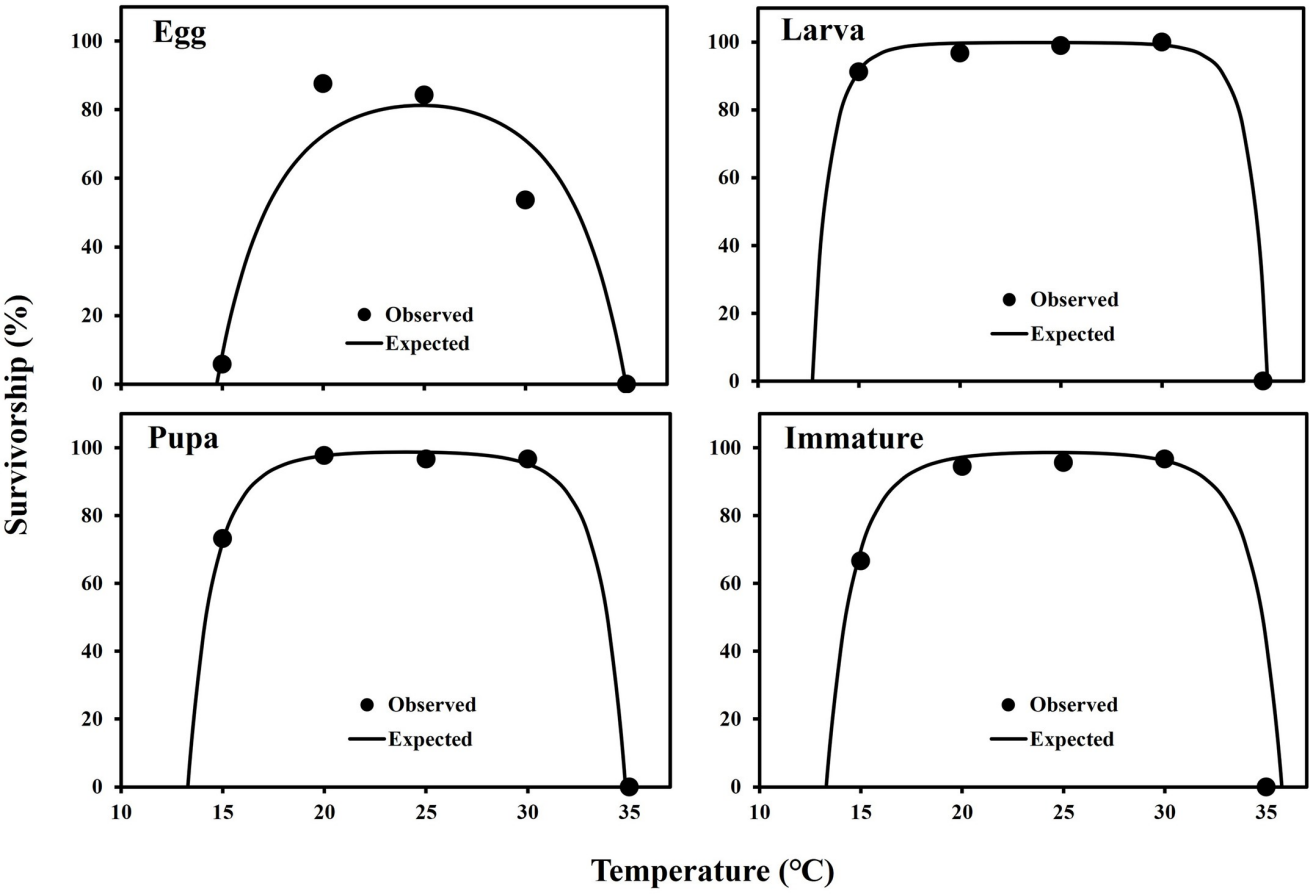
Developmental variation models of each developmental stage of *M*. *loreyi* according to accumulated degree-days.

Developmental variation models well estimated the timing of developmental completion of each stage: egg (*F* = 443.8; df = 1, 20; *P* < 0.001), larva (*F* = 2,595.1; df = 1, 67; *P* < 0.001), pupa (*F* = 1,540.4; df = 1, 40; *P* < 0.001), and immature (*F* = 3,050.2; df = 1, 75; *P* < 0.001) (Fig 3, Table 5).

**Table 5:** Parameters (estimated parameters ± SEM) of developmental variation models of *M*. *loreyi*.

Stage	Parameters		*r*^2^ value
	*α*	*β*	
Egg	53.517 ± 0.4290	18.7621 ± 4.2048	0.98
Larva	347.6 ± 1.3594	13.4229 ± 0.9585	0.99
Pupa	168.1 ± 0.8603	10.5043 ± 0.7285	0.99
Immature*	516.0 ± 1.4542	17.4875 ± 1.0955	0.99

* Immature period indicates the period from the first instar to adult emergence.

Temperature had a significant (*P* < 0.05) effect on the pre-oviposition period, longevity, and fecundity of female adults of *M*. *loreyi*, but it did not affect their post-oviposition period (Table 6). As temperature increased, the pre-oviposition period and oviposition longevity of its female adults became shorter (Table 6). However, the oviposited egg number was the highest at 25°C among the experimental temperatures in this study (Table 6).

**Table 6:** Pre-ovipositional period (day, mean ± SE), post-ovipositional period (day, mean ± SE), longevity (day, mean ± SE), and fecundity (mean ± SE) of female *M*. *loreyi* adults at constant temperatures (°C).

Temperature (°C)	Pre-oviposition	Post-oviposition	Longevity	Fecundity
15	8.9 ± 0.99 a*	2.9 ± 1.36 a	13.7 ± 1.47 a	129.4 ± 35.57 c
20	3.4 ± 0.20 b	2.4 ± 0.39 a	13.8 ± 0.76 a	548.0 ± 103.96 ab
25	2.6 ± 0.19 b	0.8 ± 0.12 a	9.0 ± 0.49 b	799.5 ± 93.58 a
30	2.2 ± 0.15 b	1.4 ± 0.35 a	7.0 ± 0.54 b	319.4 ± 79.95 bc

* Means within a column followed by the same letter are not significantly different (*P* > 0.05; Tukey’s HSD test at 95% confidence intervals).

**Fig 4:**
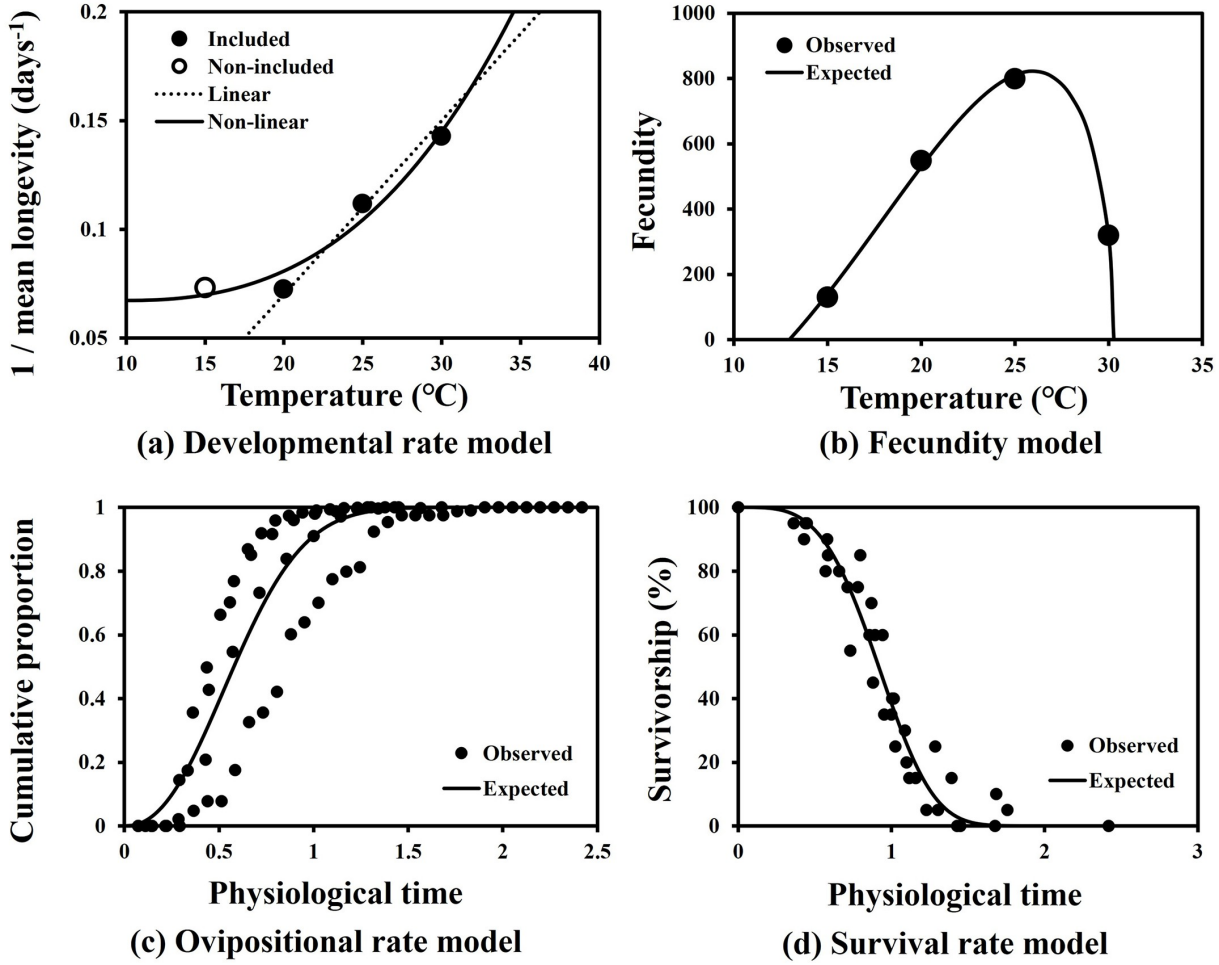
Oviposition models of *M*. *loreyi* at constant temperature (°C). (a) developmental rate model, (b) fecundity model, (c) oviposition rate model, (d) survival rate model.

Developmental rate (1 / mean longevity) of female adults of *M*. *loreyi* were well described with linear (*F* = 211.6; df = 1, 2; *P* = 0.005) and non-liner models (*F* = 23.8; df = 2, 2; *P* = 0.040) (Fig 4a, Table 7). From the linear model, the estimated LDT and thermal constant were approximately 11.3°C and 125 DDs, respectively. Fecundity over tested thermal range was well fitted with the Briére function (*F* = 588.3; df = 2, 2; *P* = 0.030) (Fig 4b, Table 7). Temperature with the maximum fecundity was expected to be 26.1°C. (Fig 4b). Oviposition rate and survival rate models over standardized physiological time of female adults were also well described by the Weibull function (Fig 4c and 4d, Table 7): oviposition rate model (*F* = 1,151.7; df = 1, 78; *P* < 0.001), and survival rate model (*F* = 785.8; df = 1, 36; *P* < 0.001).

**Table 7:** Parameters (estimated parameters ± SEM) of oviposition models of *M*. *loreyi*.

Models	Parameters	Estimated values	*r*^2^ value
Developmental rate model (non-linear)	*A*	0.07042608 ± 0.04158428	0.96
	*B*	-8.70×10^−5^ ± 2.5557×10^−4^	
	*C*	5.6607×10^−6^ ± 7.2286×10^−6^	
Developmental rate model (linear)	*a*	0.008 ± 0.0012	0.99
	*b*	-0.090 ± 0.0287	
Fecundity model	*n*	1.1722 ± 0.0622	0.99
	*T*_*min*_	12.9668 ± 0.4105	
	*T*_*max*_	30.2831 ± 0.0497	
Oviposition rate model	*α*	0.6805 ± 0.0242	0.97
	*β*	2.2772 ± 0.2592	
Survival rate model	γ	1.0120 ± 0.0159	0.98
	δ	3.6951 ± 0.3322	

### Forecasting model of second occurrence peak of *M*. *loreyi*

**Fig 5:**
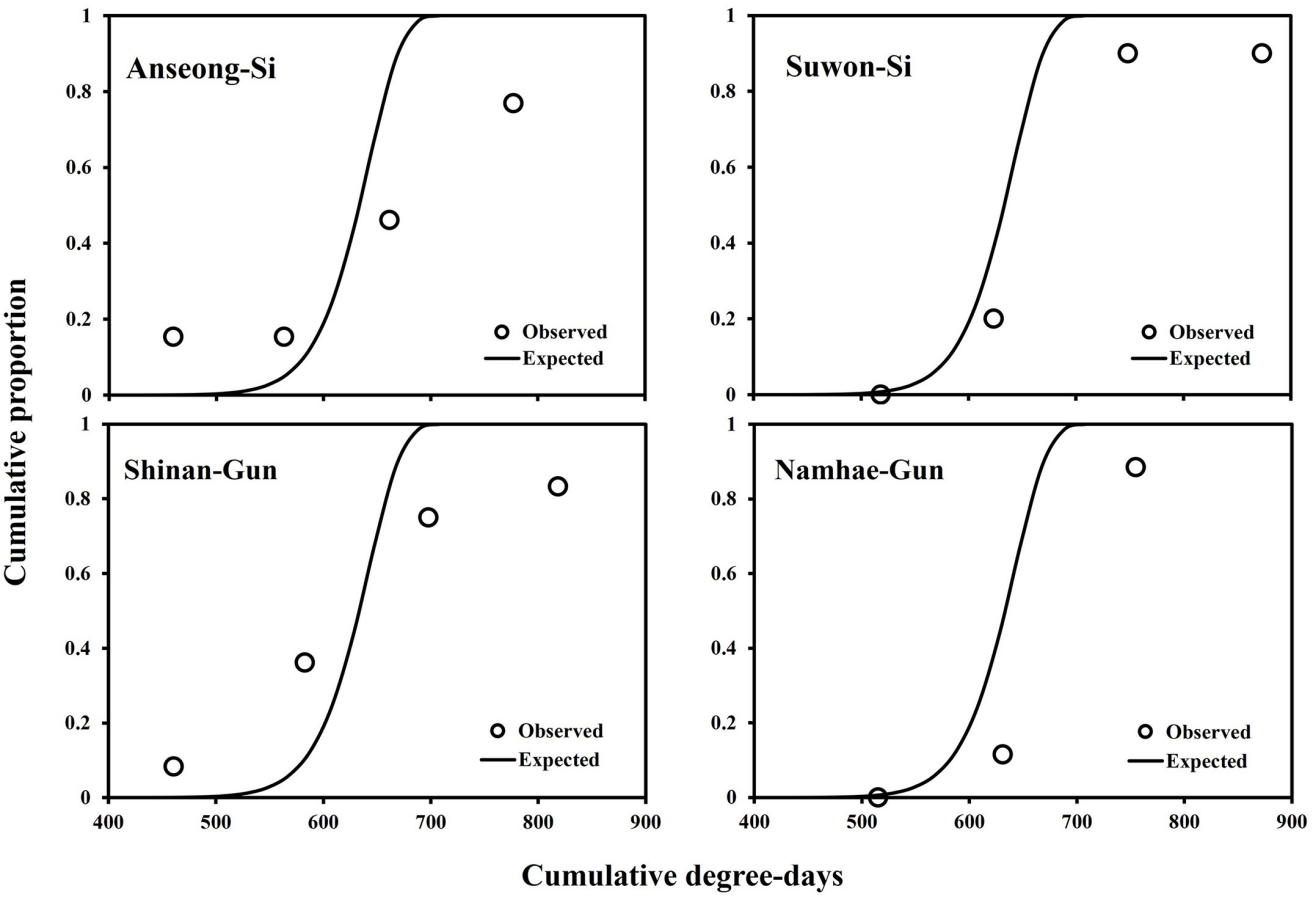
Predicted and actual second occurrence of *M*. *loreyi* adults.

Multiple steps were taken to develop the occurrence model of the second peak of *M*. *loreyi* at field conditions. First, estimated thermal requirements of 50% egg laying by female adults and egg developmental completion were calculated as ascribed at previous section in this study. The thermal requirement for 50% oviposition by female adults was 72.5 DDs by multiplying the physiological time (0.58 from the oviposition rate model) of female adults at 50% oviposition with the thermal requirement (125 DDs) estimated at adult developmental rate model. The thermal requirement of egg developmental completion was 55.6 DDs from the linear model of egg developmental model. The final model was developed by shifting the developmental variation model of *M*. *loreyi* immatures with these two thermal requirements (128.1 DDs) (Fig 5).

The developed model aptly predicted actual occurrence at two sites (Anseong-Si and Shinan-Gun) but failed to accurately predict occurrence at two sites (Suwon-Si and Namhae-gun) (Fig 5). The differences at well-predicted sites between the predicted and observed dates at 50% cumulative occurrence were one and three days at Shinan-Gun and Ansung-Si, respectively.

## Discussion

Temperature is a critical factor in the development and oviposition of *M*. *loreyi*. As temperatures increased, immature development accelerated, and adult longevity decreased. However, a decline in the survivorship of immatures and adult fecundity was noted beyond peak levels. Notably, *M*. *loreyi* eggs exhibited a higher LDT and a more limited temperature range for optimal survival compared to other stages, which differs from patterns observed in other insect species [16, 17, 26–28]. These developmental traits, coupled with flight capability [10, 12], might contribute to the species’ migratory response to colder winter temperatures.

This high LDT of *M*. *loreyi* eggs was not found in previous studies [12, 13] even though the other results (thermal requirements of all developmental stages and LDT of the other developmental stages) were similar to those of this study. Hirai [12] and Qin et al. [13] reported the LDT of its eggs as 10.5°C and 8.83°C, respectively. Both studies [12, 13] provided corn leaves as food for the immatures of *M*. *loreyi*. However, this study used an artificial diet. Moreover, the ranges of tested temperatures were 15–30°C, 18–30°C, and 15–35°C in the studies of Hirai [12], Qin et al. [13], and this study, respectively. This differences in food and tested temperature range could cause the differences in the LDT of its eggs. However, the LDT values of the previous studies [12, 13] were too low to explain the migratory behaviors of *M*. *loreyi* and to be compared to other insects [16, 17, 26–28]. Moreover, previous studies [12, 13] used fewer than 100 eggs compared to more than 2,000 eggs to estimate LDT in this study. By considering the replication number and ecological characteristics of *M*. *loreyi*, the high LDT of its eggs, 12.9°C, would be more reliable than 10.5°C or 8.83°C.

This study presented multiple temperature-dependent developmental and ovipositional models for *M*. *loreyi*. These models enhance our understanding of the species’ ecology and provide a foundation for field occurrence predictions [18], simulations of population dynamics under climate change [14, 15], and assessments of potential establishment in new regions [29]. With climatic data, these applications should be possible from local to global scales. This study showed a theoretical model developed by using these developmental and ovipositional models to predict its occurrence of second generation at field conditions. Even though this model showed high prediction accuracy at only two sites among four sites, this study proved the possibilities that the prediction model developed based on laboratory experiments could also predict the field occurrences of a certain generation of migratory insects.

The developed theoretical model in this study used the peak occurrence timing of male adults of first *M*. *loreyi* generation, required degree-days for adult 50% oviposition and egg development, and the developmental variation models of its immatures to predict its occurrence of second generation. The peak timing in the trapped adult number in the first generation at each site was used as the bio-fix to start to accumulate degree-days for prediction of the adult occurrence of its second generation. Because they would not overwinter in Korea [1, 5], the prediction of its phenology could start to cumulate degree-days when they migrated into Korea from other countries. Moreover, introduced adult insects could mate and establish populations only when the population number is over its Allee threshold [9], which is not yet known for *M*. *loreyi*. Therefore, we selected the peak timing of adult occurrence as the bio-fix of *M*. *loreyi* with a small number of captured adults of its first generation. The reasons for using the thermal constant required 50% oviposition instead of the oviposition rate model were that adults attracted to the traps would be young because they are more likely to be attracted to *M*. *loreyi* sex-pheromone and an adult number less than the Allee threshold would struggle to mate and oviposit. The oviposited eggs need a thermal requirement to complete its development. To add this thermal requirement of egg development, both the thermal constant and the developmental variation model of its eggs could be applied. In this study, the thermal constant was used to simplify the final model and focus the peak timing (50% cumulative occurrence) of adult occurrence of the second generation. The management timing of moths could be estimated from the peak occurrence of its adults by subtracting the thermal requirements of immatures [30]. The distribution of adult occurrence of *M*. *loreyi* was expressed by applying the developmental variation models of immatures.

Even in two sites showing high prediction accuracy at 50% cumulative occurrence of *M*. *loreyi*, the expected distribution of adult occurrence was much narrower than the actual distribution. This could be because component models were developed by using constant temperatures [18] and thermal constant for egg development was used instead of the developmental variation model for eggs. In the other sites, the cumulative degree-days at the actual 50% occurrence of adults were much higher than the predicted one. Continuous migration from other countries might have caused this result. In their origin countries, developed individuals would need wind currents to migrate to these sites. The time needed for suitable wind currents to form and for the individuals to fly from their origin to these sites would shift the distribution patterns of adult occurrence.

This study developed multiple models which would be helpful not only to understand the ecology of *M*. *loreyi*, but also to develop management strategies for it. Especially, the newly suggested LDT of *M*. *loreyi* could explain their migratory behaviors as an overwintering strategy. Moreover, the developed theoretical model could accurately predict the occurrence of *M*. *loreyi* second generation. This indicates that the migrated populations of *M*. *loreyi* could develop their next generation in introduced areas. Otherwise, their occurrence should be later than the predicted time. By applying the developed model in multiple points over a few years, we could determine areas more likely to be affected by migrated populations of *M*. *loreyi*. To prevent the economic damage by *M*. *loregyi* in these areas, monitoring systems should be applied and equipment to block immigration of *M*. *loreyi* populations is required because its occurrence would not be predictable. This information would be also helpful to find origin areas of *M*. *loreyi* in other countries. The findings in this study would contribute to the successful management of *M*. *loreyi*.

## Supporting information

S1 File Full data of this study. (XLSX)
